# Expression and mutational analysis of c-CBL and its relationship to the MET receptor in head and neck squamous cell carcinoma

**DOI:** 10.18632/oncotarget.9640

**Published:** 2016-05-26

**Authors:** Cleo E. Rolle, Yi-Hung Carol Tan, Tanguy Y. Seiwert, Sapana Vora, Rajani Kanteti, Rifat Hasina, George B. Carey, Mosmi Surati, Ralph R. Weichselbaum, Mark W. Lingen, Everett E. Vokes, Ravi Salgia

**Affiliations:** ^1^ Department of Medicine, The University of Chicago, Chicago, IL, USA; ^2^ Department of Pediatrics, The University of Chicago, Chicago, IL, USA; ^3^ Pritzker School of Medicine, The University of Chicago, Chicago, IL, USA; ^4^ Department of Radiation and Cellular Oncology/Ludwig Center for Metastasis Research, The University of Chicago, Chicago, IL, USA; ^5^ Department of Pathology, The University of Chicago, Chicago, IL, USA; ^6^ Department of Medical Oncology and Therapeutic Research, City of Hope, Duarte, CA, USA

**Keywords:** c-CBL, MET, head and neck cancer

## Abstract

MET is frequently overexpressed in head and neck squamous cell carcinoma (HNSCC) and degraded by c-CBL E3-ubiquitin ligase. We investigated genetic variations of c-CBL in HNSCC and the relationship between c-CBL and MET expression. High MET, low c-CBL expression was detected in 10 cell lines and 73 tumor tissues. Two novel mutations (L254S, L281F), and the single nucleotide polymorphism (SNP) P782L were identified from archival tumor tissues. 27.3% of loss of heterozygosity was found at *CBL* locus. Ectopic expression of wild-type c-CBL in SCC-35 cells downregulated MET expression and decreased cell viability. These results suggest MET overexpression is related to altered c-CBL expression, which may influence tumorigenesis.

## INTRODUCTION

It is projected that 52,610 new cases of head and neck cancer (HNC) will be diagnosed in the United States in 2014, accounting for just over 3% of estimated US cancer diagnoses [1]. Of these HNC diagnoses, it can be expected that over 90% will fall into the histologic subtype squamous cell carcinoma (HNSCC) [2]. If diagnosed early enough, HNSCC can be treated with curative intent using surgery, chemotherapy, and/or radiotherapy; nevertheless, at present, 55 to 65% of HNSCC patients will eventually die from their disease, due in part to limited treatment options for recurrent/metastatic disease [3]. To address this poor prognosis, recent research has focused on identifying molecules that can serve as therapeutic targets. Most notably, this research has produced the monoclonal antibody cetuximab [4,5], which targets the epidermal growth factor receptor (EGFR) and confers a two month overall survival (OS) advantage when combined with standard chemotherapy [6]. It is important to note that only 4-21% of patients will respond to a single agent EGFR inhibitor [6,7,8], therefore these agents are commonly combined with chemotherapy [9] or radiation therapy [4,10]. Dispite this advancement, the median OS for patients with recurrent/metastatic HNSCC has remained low at six to ten months, necessitating further research to identify molecular targets which may lead to improved therapeutics and patient outcomes [2].

One molecular target of interest is the receptor tyrosine kinase (RTK) MET, which is involved in cell motility, cell scattering, and proliferation, thus contributing to angiogenesis, invasion, and metastasis in cancer cells. As we showed in 2009, MET is highly expressed, amplified, and mutated in HNSCC; moreover, we demonstrated that MET inhibition abrogated cell proliferation and migration/motility, suggesting that MET would be an effective target in HNSCC [3]. However, we recently reported the acquisition of resistance to MET inhibition in a gastric cancer patient, despite an initial complete response [5]. This report highlights the need to improve our understanding of the underlying tumor biology of HNSCC as it relates to the regulation of MET expression and/or signaling. For this reason, we chose to investigate Casitas B-lineage lymphoma (CBL), as we found that loss of c-CBL enhanced cell viability and motility not due to EGFR, implicating c-CBL regulation of MET [11].

The mammalian CBL gene is located on human chromosome 11q23.3 [12,13]. CBL proteins belong to the RING finger class of ubiquitin ligases (E3) and have three homologues: *c-CBL*, *CBL-b*, *CBL-3* [13]. c-CBL consists of four regions encoding four functionally distinct protein domains: the N-terminal tyrosine kinase binding (TKB) domain, the catalytic RING finger domain, the proline-rich region, and the c-terminal ubiquitin-associated (UBA) domain, which also overlaps with a leucine-zipper (LZ) domain [13,14]. The TKB and RING finger domains are essential for ubiquitination of RTKs following ligand binding. The RING finger domain is required for the recruitment of E2 ubiquitin-conjugating enzymes. Included within the TKB domain are a four-helix (4H) bundle, a calcium-biding EF hand, and a modified SH2 domain, which binds to phosphotyrosine residues on activated receptors.

In the case of MET, the binding of its cognate ligand hepatocyte growth factor (HGF) activates the receptor, which is characterized by receptor dimerization and phosphorylation of tyrosine residues within the juxtamembrane (JM) domain (see Sattler et al. for review [15]). c-CBL is recruited to the receptor and initiates the cascade of MET degradation via the lysosomal-mediated pathway [16]. Abella et al. have shown that ubiquitin-deficient MET (Y1003F mutation in the JM domain) has increased stability as well as enhanced oncogenic activity *in vivo* [17]. In HNSCC, it may be that the pathogenesis of these tumors is linked to the overexpression of MET, which is caused by dysregulated receptor cycling via c-CBL. Therefore, we sought to determine the expression and mutational status of c-CBL in HNSCC, as well as its relationship to MET expression.

In the investigation described below, we observed low expression of c-CBL and moderate to high expression of MET in HNSCC tumor tissues. Similar findings were obtained by immunoblot analysis of HNSCC cell lines. Moreover, we identified two novel somatic mutations (L254S, L281F) in the TKB domain of c-CBL. Loss of heterozygosity (LOH) analysis of the *CBL* locus in tumor/normal pairwise tissues revealed significant LOH (27.3%, n=6/22). Taken along with our previous studies of MET in HNSCC, our investigation suggests that *CBL* mutations can occur exclusive of *MET* mutations [3]. We thus hypothesize that altered c-CBL expression contributes to the oncogenic potential of MET largely through dysregulated processing of activated MET in HNSCC. Thus, we believe that we have identified an unexpected relationship between c-CBL and enhanced MET signaling in HNSCC and that c-CBL expression may therefore serve as a potential biomarker for the selection of patients that would benefit the most from MET inhibitor treatment.

## RESULTS

### Expression of c-CBL and MET in HNSCC

**Figure 1:**
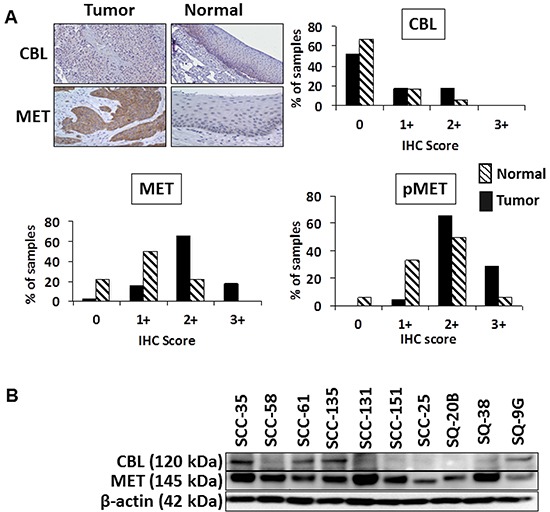
Expression of c-CBL and MET in HNSCC tumor specimens **A**. Representative IHC images of immunostained TMAs for the expression of c-CBL and MET in HNSCC tumor specimens. Analysis of the distribution of IHC scores for c-CBL, MET, and pMET (Y1230/34/35) for adjacent normal (n=18) and tumor cores (n=73). TMAs were scored on a scale from 0 (no staining/no protein expression) to 3+ (strong staining/high protein expression). **B**. Whole cell lysates from 10 HNSCC cell lines were subjected to SDS-PAGE, then immunoblotted using the indicated antibodies. β-actin served as the loading control.

In a previous study we determined MET to be highly overexpressed and often mutated in HNSCC [3]. Since c-CBL regulates the processing of MET, we sought to determine whether the overexpression of MET was due to altered expression of c-CBL. For this study we focused our analysis on adjacent normal (n=18) and primary tumor (n=73) cores contained within a TMA that was subjected to immunohistochemistry (IHC) staining to determine the relationship between c-CBL, MET, and phosphorylated MET (Y1230/34/35 and Y1003). Representative IHC staining of normal and tumor specimens for the expression of c-CBL and MET are shown in Figure 1A. Each of the tumor and normal cores contained in the TMA were scored independently by two pathologists on a 4-point scale of 0 to 3+ as described in the Materials and Methods section. The distribution of IHC scores are summarized in Figure 1A. c-CBL expression was low or undetectable in both normal (0=66.7%, 1+=16.7%, 2+=5.6%, and 3+=0%) and tumor tissues (0=52.1%, 1+=17.8%, 2+=17.8%, and 3+=0%). The mean c-CBL scores were not significantly different between normal (0.294±0.142) and tumor tissues (0.61±0.101). In contrast, MET expression was elevated in tumor tissues (0=1.4%, 1+=15.1%, 2+=65.8%, and 3+=17.8%) relative to normal tissues (0=22.2%, 1+=50%, 2+=22.2%, and 3+=0%). The mean score for MET in tumor tissues (1.993±0.07) was significantly higher (p=0.001) than that of normal tissues (1.0±0.17). The mean pMET (Y1003) expression was significantly higher (p=0.0003) in tumor tissues (2.25±0.06) compared to normal tissues (1.588±0.17). The mean IHC score for pMET (Y1230/34/35) was also significantly higher (p=0.02) in tumor tissues (0.71±0.075) compared to normal tissues (0.3125±0.12).

To further evaluate the relationship between c-CBL and MET in HNSCC, we analyzed the expression of MET and c-CBL in a panel of HNSCC cell lines (Figure 1B). MET was expressed at moderate to high levels in 10/10 cell lines assayed, with the highest expression detected in SCC-35 and SCC-131 cells. c-CBL expression was low or undetectable in all cell lines, with SCC-35 cells having the highest expression. Four cell lines expressed MET and low levels of c-CBL: SCC-35, SCC-61, SCC-135, and SQ-9G. Six cell lines expressed MET in the absence of c-CBL: SCC-58, SCC-131, SCC-151, SCC-25, SQ-20B, and SQ-38. These findings largely recapitulated our findings in primary tumor specimens, such that highest expression of MET was detected in the cell lines with the lowest expression of c-CBL.

### *CBL* gene mutations in HNSCC

**Figure 2:**
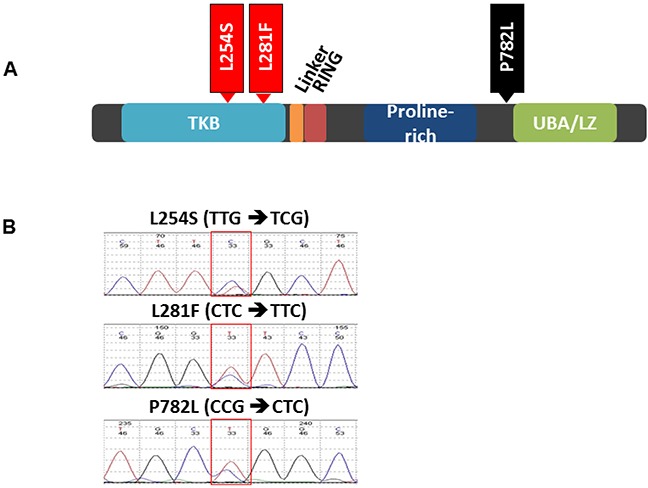
*c-CBL* mutations in HNSCC **A**. Schematic of the functional domains of the c-CBL protein and the location of the mutations identified in 2/20 HNSCC tumor specimens. **B**. Representative sequencing chromatograms of the mutation region.

Previously, we have shown somatic mutations in *CBL* in lung cancer [11]. To determine the mutational status of *CBL* in HNSCC, genomic DNA was isolated from FFPE patient tumor specimens (n=20) and HNSCC cell lines (n=16). No *CBL* mutations were detected in the cell lines. However, we identified 3 missense mutations among three different patients (Figure 2A). The known single nucleotide polymorphism (SNP) in exon 15 (P782L, rs2229073) was detected in one sample. Importantly, we identified two novel mutations in exon 5 (L254S and L281F). The mutations L254S and L281F were localized to the TKB domain (Figure 2B).

### LOH of CBL gene

**Figure 3:**
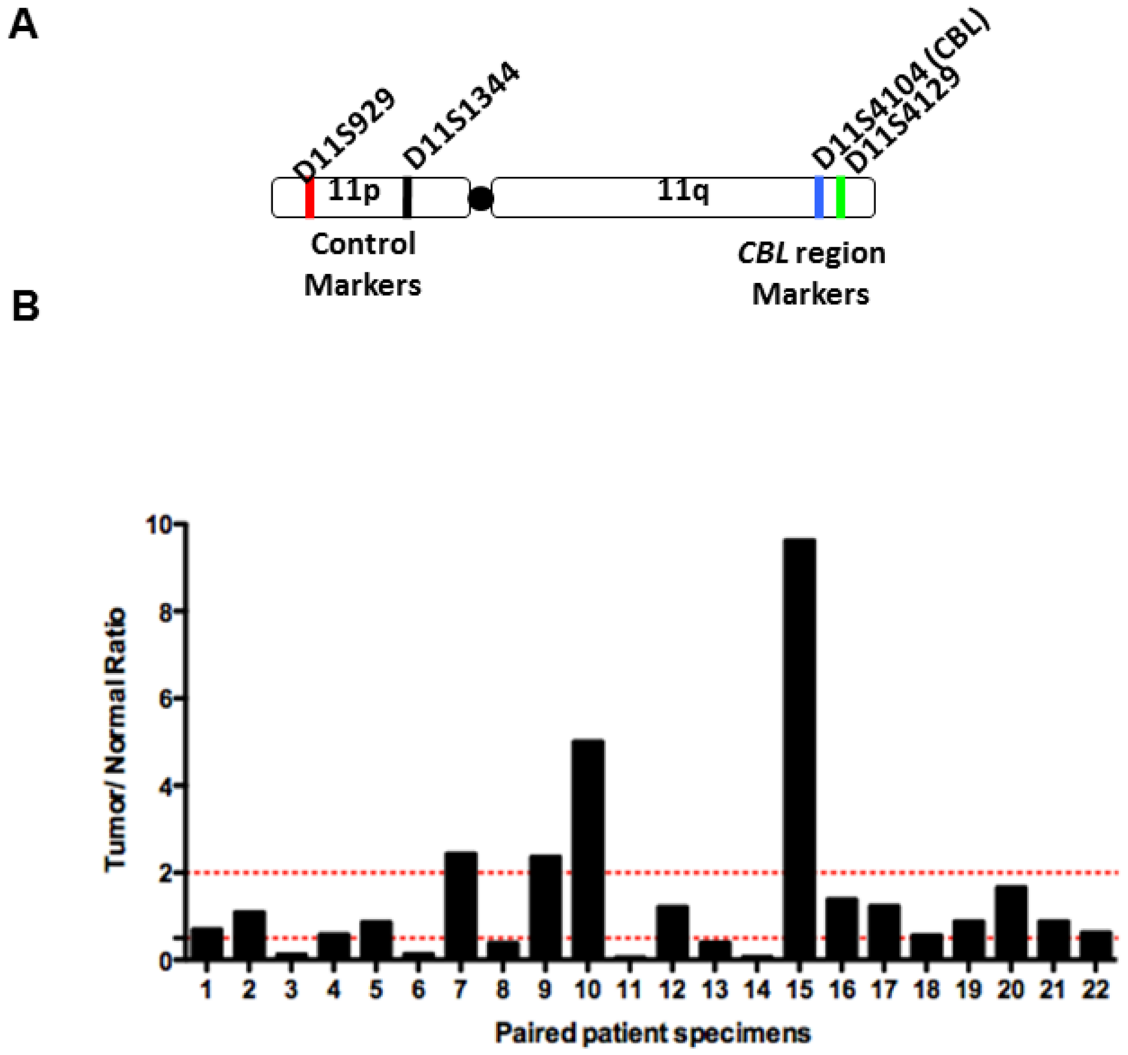
LOH at the *c-CBL* locus in HNSCC LOH analysis of 23 tumor and paired normal patient samples was conducted. After PCR amplification using chromosome 11 specific microsatelliteprimers, the PCR product was separated by capillary electrophoresis and bands were quantified according to intensity. **A**. Schematic of chromosome 11 with location of primers and representative examples of LOH chromatogram analysis. **B**. Summary bar graph of LOH results. A ratio of Tumor:Normal<0.5 indicates LOH at the c-CBL locus.

LOH at the *CBL* locus was assayed in 25 paired normal and corresponding HNSCC tumor samples. LOH was detected in 6/22 (27.3%) samples, based on microsatellite markers within the *CBL* locus and 400bp upstream of the *CBL* locus on 11q, as well as two control markers on 11p (Figure 3A, 3B). Normal allelic contribution was detected in 12/22 (54.5%) samples. In two samples we determined gross increased genetic material (n=2/22, 9.1%). We were unable to determine the LOH status of 3 paired samples.

### Ectopic expression of c-CBL decreases MET expression and cell viability

**Figure 4:**
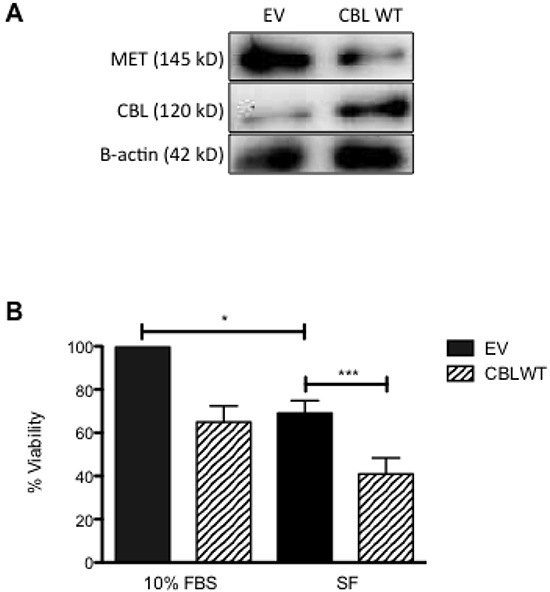
The effect of ectopic expression of c-CBL WT on cell viability HNSCC cells were maintained in media or transiently transfected with empty vector (EV) and vector containing wild-type c-CBL (c-CBL WT). **A**. At 48h post-transfection, cells were harvested to assess c-CBL protein expression by immunoblot (IB). **B**. Cells were cultured in serum-free media for 24h then cell viability was determined by MTT assay. Cell viability was normalized to EV transfected cells.

Having shown an inverse relationship between c-CBL and MET expression in HNSCC, we then investigated the cellular functionality of this relationship. SCC-35 and SCC-131 were transiently transfected with empty vector (EV) or a vector containing c-CBL wild-type (c-CBL WT). At 48h post-transfection, the expression of c-CBL and MET was determined by immunoblotting. Expression of c-CBL was confirmed in the cells transiently transfected with c-CBL WT, but not in those transfected with EV (Figure 4A). By using Immunoprecipitation, the result also confirmed that MET and c-CBL were co-localized (data not shown). Concomitant to c-CBL expression was downregulation of MET expression. The cell viability of transiently transfected cells was determined at 48h post-transfection by standard MTT assay (Figure 4B). Ectopic expression of c-CBL WT in SCC-35 decreased the cell viability relative to EV transfected cells. Similar results were obtained using c-CBL WT transfected SCC-151 cells (data not shown).

## DISCUSSION

c-CBL protein expression is low in normal and HNSCC patient tumor specimens, while MET and pMET expression are increased. This pattern of c-CBL and MET protein expression was largely recapitulated in HNSCC cell lines. In HNSCC tumor specimens, *CBL* was mutated and LOH was detected at the *CBL* locus. Additionally, both our current data, as well as our previous publication clearly demonstrate that MET is activated in HNSCC, and pMET expression was concomitant with MET expression [3]. Taken together, these data support the notion that the low levels of c-CBL detected in HNSCC is not sufficient to control the expression of MET.

c-CBL has been shown to regulate the expression of a number of proteins such as RTKs and other signal transduction molecules [13]. Herein, in HNSCC we specifically demonstrate that c-CBL expression is low to not detectable. Moreover, we determined LOH at the *CBL* locus. It is important to note that in certain hematologic malignancies *CBL* shows uniparental disomy with activating *CBL* mutations [22]. This was not detected in our current study of HNSCC or previously in non-small cell lung cancer [11]. Therefore, suggesting that there are potential established differences between hematological malignancies and solid tumors, namely upper aerodigestive malignancies. Interestingly, the relatively low expression level of c-CBL would suggest that this molecule functions as a tumor suppressor in HNSCC. However, in other tumors it likely functions as an adaptor molecule, in which case we have shown previously that BCR/ABL utilizes c-CBL for a plethora of signal transduction [23,24].

MET is largely overexpressed in HNSCC and we have shown that it can be effectively targeted using small molecule chemical inhibitors [3]. To date, there are a number of drugs and anti-MET antibodies that have come to clinical trials, including MetMAb, crizotinib, XL184, and ARQ 197. However, not all these MET inhibitors demonstrated promising therapeutic outcomes in solid tumors. In our previous studies in lung cancer, the results not only showed the interation of c-CBL and MET [11] but also showed knockdown c-CBL induced MET overexpression and became sensitive to MET inhibitors (data not shown). Moreover, Tarceva resistance lung cnacer cells became sensitive to MET inhibitors after knocking down c-CBL (data not shown). Head and neck cancer is quite different than lung cancer. Majority of HNSCC patients are HPV positive and have different response to the therapy compared to lung cancer. It would be important to study anti-MET therapeutics and determine the functional role of c-CBL in HNSCC to identify better biomarkers of response to MET targeted therapies. It is very possible that c-CBL could be a predictive marker for anti-MET therapeutics since low levels of c-CBL is linked to high expression of MET. It is important that further studies are needed to identify the effect of the interaction between *MET* mutations and c-CBL expression or vice versa. As has been previously reported, the JM domain of MET binds to c-CBL, which leads to its degradation [25,26]. Our laboratory has identified gain-of-function JM domain mutations, including exon 14 skipping, in MET that we predict will alter its binding to c-CBL.

The two novel mutations that we identified in HNSCC were localized to the TKB domain of c-CBL. The TKB domain is required for binding and ubiquitination of MET [27]. The TKB domain of c-CBL binds the Y1003 residue of MET. Alternatively, c-CBL can also interact with MET via the adaptor protein Grb2, which binds the Y1356 residue in MET and the proline-rich domain of c-CBL. In the TCGA database, there were 279 head and neck cancer tumors have been tested. only two mutations (A208P and E765K) and *CBL* deletions were reported [28]. Based on our mutational data it is likely that the mutations that we detected in *CBL* were responsible for the enhanced expression of MET in HNSCC, since we determined high expression of Y1003 in our primary tumor specimens. Although further studies are necessary to characterize the function of the identified TKB mutants in HNSCC, we believe that our data supports the notion that the overexpression of MET is likely due to dysregulated receptor processing as a result of impaired c-CBL activity in the L254S and L281F mutants. Our *in vitro* studies using HNSCC cell lines revealed a concomitant decrease of MET and cell viability upon ectopic CBL expression. The mechanisms underlying the c-CBL-mediated downregulation of MET and its impact on cell viability in HNSCC need to be investigated further, as well as the role of these newly identified mutations.

In summary, c-CBL expression is low, and at the gene level *CBL* is mutated and has LOH in HNSCC. In tumor tissues, low c-CBL expression was concomitant to high MET and pMET expression. It would now be useful to determine the role of c-CBL as a prognosticator and/or predictive biomarker in HNSCC.

## MATERIALS AND METHODS

### Ethics statement

This human subjects research was approved by the Institutional Review Board (IRB) at The University of Chicago and covers all research conducted in the laboratory. Archived tissues were obtained under consent-waived protocols for de-identified patients (10-653N) or deceased patients (10-654N). The IRB can be contacted at The University of Chicago, McGiffert Hall, 5751 S. Woodlawn Ave., 2^nd^ Floor, Chicago, IL 60637.

### Tissue microarray and immunohistochemistry

Upon approval from the IRB, tissue blocks for the study were selected from patients with HNSCC who were treated at the University of Chicago Medical Center (diagnosed between 1992 and 2005). The tissue microarrays (TMA) used in this study were composed of 73 tumor cores, 12 dysplasia cores, and 18 adjacent normal cores derived from 41 patients. TMAs were built using the ATA-27 Arrayer from Beecher Instruments (Sun Prairie, WI) as previously described [18]. In brief, tissue cores (1.5-mm punch) were organized precisely into a grid and embedded in paraffin. TMA slides were deparaffinized and antigen recovery was performed as previously described [19]. Slides were incubated with BSA to block nonspecific staining, followed by immunostaining using anti-CBL (rabbit polyclonal, Abcam, Cambridge, MA), anti-MET (mouse monoclonal, Invitrogen, Carlsabad, CA), and anti-pMET([Y1003] mouse monoclonal and [Y1230/34/35] rabbit polyclonal, Invitrogen, Carlsbad, CA) antibodies. The corresponding secondary antibodies, goat-anti-rabbit or goat-anti-mouse IgG, conjugated to HRP-labeled polymers (Envision+ System, DAKO, Carpinteria, CA) were used to detect antibody binding and the proteins were visualized using diaminobenzidine chromogen (DAKO). TMA slides were counterstained with hematoxylin. Negative controls for immunostaining were prepared by substituting the primary antibodies with non-immune mouse or rabbit serum. All slides were reviewed and scored independently by two pathologists, and if the two scores were discordant the pathologists conferred until a consensus was reached. As previously described [18], the relative intensity of the immunostaining was scored using a 0 to 3+ scale, with 0 denoting no staining/no protein expression, 1+ denoting weak positive staining/low protein expression, 2+ denoting moderate positive staining/moderate protein expression, and3+ denoting strong positive staining/high protein expression.

### Cell lines and culture

The cell lines SCC-35 [19,20], SCC-61 [19,20], SCC-131 [21], SCC-135 [19], SCC-151 [19], SQ-9G [20], SQ-20B [19,20], and SQ-38 [20] were obtained from Dr. Ralph Weichselbaum (The University of Chicago). SCC-25 and SCC-58 [21] were obtained from Dr. Mark Lingen (The University of Chicago). The cells were maintained in DMEM/F12 medium containing penicillin/streptomycin and supplemented with 10% FBS.

### Immunoblotting

Cells were harvested and washed in 1X phosphate buffered saline (PBS), then lysed in ice-cold M-PER lysis buffer plus HALT protease and HALT phosphatase inhibitors (Pierce, Rockford, IL). Total cell lysates (50 μg/well) were separated by SDS-PAGE electrophoresis and transferred onto immobilon-P membranes (Whatman, Piscataway, NJ). Membranes were blocked with 5% BSA in Tris-buffered saline containing Tween-20 (TBST) (1x TBS, 0.1% Tween-20) for 1h at room temperature and incubated with the appropriate primary antibody at 4°C overnight. Membranes were then washed three times with TBST and probed with the appropriate horseradish peroxidase (HRP)-conjugated secondary antibody. The membranes were again washed three times in TBST and bands were visualized using Western blot chemiluminescence reagent (BioRad, Valencia, CA) on a Chemidoc Gel documentation system (BioRad). The following antibodies were used: c-CBL (Abcam, Cambridge, MA); MET (Invitrogen, Carlsbad, CA); and β-actin (Sigma, St. Louis, MO).

### c-CBL gene mutational analysis

Genomic DNA was isolated from formalin-fixed, paraffin embedded (FFPE) patient tissues using the QIAamp DNA Minikit (Qiagen, Venlo, Netherlands) according to the manufacturer's instructions. Exons 2 to 16 of c-CBL were amplified and sequenced as previously reported [11]. The PCR conditions were as follows: 1 cycle - 95°C for 5 min; 30 cycles - 95°C for 30 s, 58°C for 30 s, and 72°C for 1 min; 1 cycle - 72°C for 5 min. Sequencing was performed on the forward coding strand with confirmation of *CBL* alterations performed by sequencing the reverse strand. Mutational analysis was performed using Mutation Surveyor v2.61 (Softgenetics, State College, PA).

### Loss of heterozygosity (LOH) analysis

*CBL* LOH analysis was conducted as previously described [11]. Briefly, four microsatellites on chromosome 11 (two on 11q - one 200 kb upstream and one within the *CBL* gene - and two control markers on 11p) were selected for analysis (Table 1). Genomic DNA was extracted from FFPE tumor samples and paired normal tissue. Marker D11S929 served as an internal control to check for consistency in PCRs and of peaks from capillary electrophoresis. PCRs were carried out in a volume of 10 μL that contained 1 μL genomic DNA (20–50 ng), 0.5 μM of each primer (1.0 μM total for each primer pair), 400 μM dNTPs, 1X PCR buffer containing MgCl_2_, and 0.2U *Taq* DNA polymerase. The PCR conditions were as follows: 5 min at 95°C; 30 cycles of 30 sec at 95°C, 1 min at 60°C, 1 min at 72°C; and 5 min at 72°C. Peak Scanner 1.0 (Applied Biosystems, Carlsbad, CA) was used to analyze the chromatograms. The ratio of the allelic areas was calculated for each tumor and paired normal DNA sample. When the qLOH (allelic ratio for the tumor peaks divided by the allelic ratio of paired normal sample) was ≤0.5 or ≥2.0 for *CBL* and at least one other 11q marker in at least two separate experiments, the sample was considered to have an allelic imbalance and was interpreted as LOH.

**Table 1:** Primers used for *c-CBL* LOH analysis

Primer Set	Primer sequence	Dyes
1 D11S4129	5′-GGCCACTGCCCTTACCATCA-3′ 5′-ACAGCGACCACATCTCCTGC-3′	VIC
2 D11S4104	5′-GGAGAATGGCCTGAACCTG-3′ 5′-ATCTCTATCATGGGCAATTTGG-3′	6-FAM
3 D11S929	5′-CCCAGTTGCCGAACTACC-3′ 5′-AGGCCCTTCCAAGATCAG-3′	PET
4 D11S1344	5′-CCCTGAACTTCTGCATTCAC-3′ 5′-GCGCCTGGCTTGTACATATA-3′	NED

### Transient transfection of HNSCC cells

HNSCC cell lines (SCC-35 and SCC-151) were transiently transfected using Fugene HD reagent (Roche, Nutley, NJ) according to the manufacturer's instructions. Cells were transfected with 4μg of plasmid DNA containing no insert (empty vector, EV) or c-CBL wild-type (WT). After 48h cell lysates were collected for protein expression by immunoblotting.

### Cell viability assay

The cells were cultured for 24h following the transfection in complete media. Cell viability was determined using a standard MTT assay (Sigma, St. Louis, MO) by culturing the cells (1×10^5^ cells/well in a 96-well plate) for 24h in serum-free media and complete media.
